# Visible light-induced photocatalytic and antibacterial adhesion properties of superhydrophilic TiO_2_ nanoparticles

**DOI:** 10.1038/s41598-024-58660-0

**Published:** 2024-04-04

**Authors:** Mingzhu Zhou, Xingran Zhang, Yuanxia Quan, Yu Tian, Jie Chen, Li Li

**Affiliations:** School of Chemistry and Chemical Engineering, Chongqing University of Technology, Chongqing, 400054 China

**Keywords:** Anatase TiO_2_, Ti^3+^/OVs, Hydrophilicity, Photocatalysis, Anti-bacterial adhesion, Materials science, Biomaterials, Materials for energy and catalysis, Materials for optics, Nanoscale materials

## Abstract

Bacterial infections triggered by patient or healthcare worker contact with surfaces are a major cause of medically acquired infections. By controlling the kinetics of tetrabutyl titanate hydrolysis and condensation during the sol–gel process, it is possible to regulate the content of Ti^3+^ and oxygen vacancies (OVs) in TiO_2_, and adjust the associated visible light-induced photocatalytic performance and anti-bacterial adhesion properties. The results have shown that the Ti^3+^ content in TiO_2_ was 9.87% at the calcination temperature of the reaction system was 300 °C and pH was 1.0, corresponding to optimal photocatalytic and hydrophilic properties. The formation of a hydrated layer on the superhydrophilic surface provided resistance to bacterial adhesion, preventing cross-contamination on high-touch surfaces. The excellent photocatalytic self-cleaning performance and anti-bacterial adhesion properties can be attributed to synergistic effects associated with the high specific surface area of TiO_2_ nanoparticles, the mesoporous structure, and the presence of Ti^3+^ and OVs. The formation of superhydrophilic self-cleaning surfaces under visible light can serve as the basis for the development of a new class of anti-bacterial adhesion materials.

## Introduction

The outbreak of various infectious diseases, such as novel coronavirus pneumonia (COVID-19), influenza, and Ebola (EBOV), have had a significant impact on the global economy and health systems^1–3^. This was particularly evident in the case of the human novel coronavirus (SARS-CoV-2) infection, which has resulted in over 767 million cases and 6.94 million deaths^4,5^. Bacteria and viruses can be transmitted through surfaces and objects that have been touched by patients or healthcare workers,including medical equipment, furniture, doorknobs, and walls^6,7^. The associated microorganisms are a major cause of healthcare-relateded and community-acquired infections. As a result, there is a growing demand for materials that exhibit self-cleaning functions and antimicrobial effects.

In recent years, titanium dioxide (TiO_2_) has gained significant attention as an effective photocatalytic and self-cleaning material with the benefits of low cost, corrosion resistance, biocompatibility, and chemical stability.Titania is highly oxidizing when exposed to UV light, producing reactive oxygen species (ROS) such as hydroxyl (^**.**^OH), oxide (**·**O_2_^−^), and superoxide hydrogen (HO_2_^**.**^) radicals , which act on living organisms to kill bacteria, fungi, and viruses^8,9^. Moreover, exposure to UV light generates surface TiO_2_ electron–hole (e^−^–h^+^) pairs, which are conducive to the adsorption of -OH analogues, forming a superhydrophilic surface^10,11^with a resultant combination of photocatalytic properties and superhydrophilicity. The superhydrophilic surface limits bacterial adhesion, and any organic matter and bacteria attached to the titanium dioxide surface can be decomposed and inactivated through light-induced ROS generation^12–14^. However, the wide-band gap energy (3.2 eV) associated with anatase TiO_2_ means it is only responsive to UV radiation, which greatly limits practical applications^15^. In order to overcome this limitation, surface modification, use of a heterojunction, ion doping, and the introduction of oxygen defects have been proposed^16–19^. Taking an overview of this work, oxygen defect engineering and self-doping of Ti^3+^ represent the most efficient approaches. The objective is to reduce the energy band gap of TiO_2_ and also decrease the rate of photogenerated e^−^–h^+^ complexes, so that the optical absorption edge shifts from ultraviolet light to the visible region, thereby enhancing the visible light activity and stability of TiO_2_^20,21^.

Modified TiO_2_ exhibits high efficacy in inactivating a wide range of bacteria and viruses under visible light and has been widely used in dental, orthopedic, and bone implants to prevent bacterial infections^22–25^. In addition, superhydrophilic TiO_2_ with a visible light response has been applied as a coating for surfaces such as automotive glass, construction materials, and medical devices due to its enhanced self-cleaning and antifouling properties^13^. A superhydrophilic TiO_2_ surface provides resistance to bacterial adhesion, thereby effectively inhibiting the formation of biofilms. Hu et al.^26^ have proposed that the anti-bacterial adhesion mechanism of superhydrophilic materials can be attributed to the creation of a surface hydrated layer that exhibits repulsive and spatial site-barrier effects. This hydrated layer acts as a barrier, preventing bacteria from penetrating, contacting, and adhering to the material^27^. There have been few studies that address utilization of superhydrophilic properties to inhibit biofilm formation, and most studies have focused on superhydrophobic surfaces^28–30^. Superhydrophilic surfaces are more conducive than superhydrophobic surfaces to antimicrobial release^31^. Consequently, the application of self-cleaning nano-TiO_2_, which exhibits superhydrophilicity and resistance to bacterial adhesion under visible light warrants further investigation.

In this study, a superhydrophilic, strongly visible-light-responsive Ti^3+^ and OVs-doped TiO_2_ with excellent photocatalytic activity and resistance to bacterial adhesion has been developed. The crystal conformation, surface properties, and defect structure of anatase TiO_2_ nanoparticles were modulated by controlling the pH and calcination temperature. We have evaluated the ability of TiO_2_, synthesized at different pH and calcination temperatures, to degrade methyl orange (MO) under simulated sunlight (full-spectrum) and visible light radiation exposure. The mechanism of MO photocatalytic oxidation has been examined using free radical detection. In addition, we have investigated the hydrophilic properties of different TiO_2_ under natural light. The ability of the hydrophilic films to resist adhesion of E. coli was quantitatively investigated using the plate coating method. The defect structure of TiO_2_ can be controlled using a simple method to broaden its absorption spectrum, resulting in improved photocatalytic activity and antibacterial adhesion properties under visible light. The findings of this study provide theoretical support for the future application of TiO_2_ in the medical field to prevent bacterial infections caused by medical personnel coming into contact with infected surfaces.

## Materials and methods

### Preparation of anatase TiO_2_

**Figure 1:**
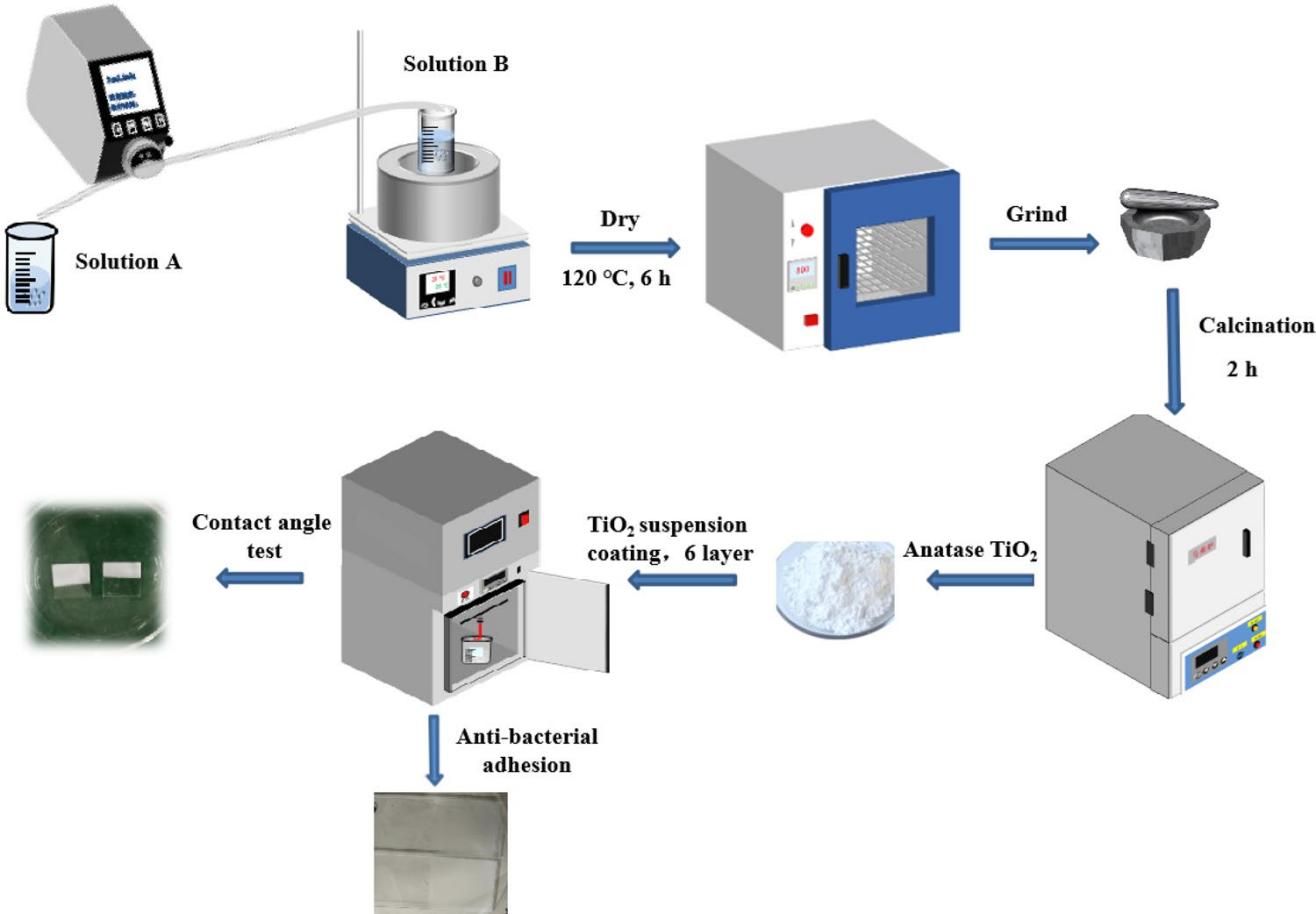
Process flow diagram for the preparation of TiO_2_ nanopowder and thin films.

Titania (TiO_2_) was synthesized using a low temperature sol–gel method, as shown in Fig. 1. Tetrabutyl titanate (TBOT) was slowly added to anhydrous ethanol to form a TBOT master-batch with a molar concentration of 0.59 M. A 24 mL sample of the TBOT master-batch was measured and labeled “solution A”. A 2 mL volume of diluted acetic acid solution (2% by mass) was added to a beaker containing 35 mL of anhydrous ethanol with hydrochloric acid (HCl) as a catalyst, and the pH was adjusted to give solution B. At a H^+^/Ti molar ratio of 0, 0.001, 0.2 and 0.5, the corresponding pH was 0.6, 1.0, 1.9 and 4.7, respectively. A peristaltic pump was used to deliver solution A dropwise into solution B at a rate of 3 mL/min. The mixture was then agitated in a water bath at 25 °C for 3 h to produce a sol, and following a 20 h aging period, a gel was formed. The resultant samples were dried at 120 °C for 6 h, and treated in air at 300, 400, 500, 600, and 700 °C for 2 h using a heating rate of 4 °C/min. Finally, the calcined samples were ground in agate to obtain TiO_2_ powder. The samples are denoted as TN-X-Y, where X represents the pH and Y represents the calcination temperature.

Titania films were also prepared. Microscope coverslips and slides were used as substrates for the TiO_2_ films, which were ultrasonically cleaned sequentially with acetone, anhydrous ethanol, and deionised water before use to remove organic and inorganic contaminants. The surfaces were then dried and made ready for use. Slides were utilized for assessing anti-bacterial adhesion whereas coverslip substrates were employed for contact angle testing. The TiO_2_ powder was dissolved in anhydrous ethanol and subjected to ultrasonication for 30 min to produce a suspension with a concentration of 13 g/L. The application of the films onto the glass substrate was performed using a constant-temperature impregnation lift coater (Shanghai Sanyan Technology, SYDC-200H) with a descending speed of 3 mm/s, lifting speed of 1 mm/s, impregnation time of 30 s, retention time of 120 s, and six coatings.

## Morphological and chemical characteristics

Surface elemental analysis was conducted using an X-ray photoelectron spectrometer (XPS, Thermo Fisher Scientific, Thermo Scientific ESCALAB Xi +). Specific surface area and pore size analyses were performed using a specific surface area tester (BET, Bayside Instrument Technology (Beijing), BSD-660S A6). Oxygen vacancy and photogenerated radical testing were carried out using electron paramagnetic resonance (EPR, Germany, Bruker EMX PLUS). The light absorption spectra of TiO_2_ in the wavelength range of 200–800 nm were measured using a UV–Vis diffuse reflectance spectrometer (UV–Vis-DRS, Shimadzu, UV-2700i). The physical phase composition of the materials was analyzed using an X-ray diffractometer (XRD, Shimadzu, XRD-7000S) over a 2θ scanning range from 20° to 80° and a scanning speed of 2°/min. The crystal size of the anatase TiO_2_ (101) crystalline faces was calculated using the Scherrer Equation^32^. The infrared spectra (IR) of the samples (prepared as KBr tablets) were obtained on a Fourier transform infrared spectrometer (IR Prestige-21, SHMADZU). Thermogravimetric analysis (TGA) of the samples was performed using a TA instrument (TGA/DSC1/1600LF, Mettler Toledo).

## Hydrophilicity test

The static water contact angle (WCA) of the TiO_2_ films was determined using a video contact angle tester (DropMeterTM A-200, Ningbo Haishu Maishu Inspection Technology Co., Ltd). A 2.0 μL droplet was placed on the TiO_2_ film using a syringe. The droplet shape was then captured using a CCD camera, and the static contact angle of water subsequently calculated. The water droplets were placed at four different locations for each sample, and the average contact angle value was determined.

## Evaluation of photocatalytic activity

A xenon lamp was used as a simulated sunlight source (full-spectrum), whereas visible light with a wavelength of 420–800 nm was employed to degrade methyl orange (MO) at a photocurrent of 15 mA. The degradation of MO by TiO_2_ samples was assessed using an ultraviolet–visible spectrophotometer (UV–Vis). The experiment was conducted at room temperature by adding 50 mg anatase TiO_2_ (0.5 g/L) to a 100 mL solution of MO with an initial concentration of 5 mg/L. The mixture of photocatalyst and MO was allowed to reach adsorption–desorption equilibrium by incubating in the dark for 60 min. During the photoreaction phase, the mixed solutions were exposed to simulated sunlight for 1 h. Samples were collected at 10-min intervals, and the concentration of MO remaining was determined by UV–Vis analysis (at 464 nm). In addition, the reaction was conducted under visible light irradiation for 2 h. Samples were taken at 20-min intervals to monitor the residual concentration of MO using the same method.

## Anti-bacterial adhesion assay

The anti-adhesion experiment was performed using the Gram-negative strain E. coli ATCC 25,922. A 3 mL sample of LB liquid medium was added to two 12 mL bacterial culture tubes. Single colonies were selected from the E. coli solid medium and transferred to the liquid medium; the second liquid medium served as a blank. The samples were placed in a constant temperature shaker (37 °C, 200 rpm) and incubated for 15 h. The bacterial solution was then diluted to 10^6^ CFU/mL using a saline solution (0.85% NaCl). A 304 μL sample of the bacterial solution was uniformly added to the sample surface. The dimensions of the glass matrix were 2.5 cm × 7.6 cm. Sterilized tweezers were used to pick up the sterilized cover film and individually cover the samples, ensuring that it was spread flat so that the bacteria touched the samples. The surfaces were evenly coated with bacteria, ensuring that they did not extend beyond the edges of the covering film. The surfaces were then incubated at 37 °C for 6 h for bacterial adhesion. After this period, the samples were carefully picked up using sterile forceps and washed three times with saline to remove any bacteria that did not fully adhere. The washed samples were transferred to new petri dishes, and 20 mL saline solution was added. Ultrasound was applied for 15 min to completely dislodge the adhering bacteria. Serial tenfold dilutions of the bacterial solution utilized sterile PBS solution. A 100 μL sample of the diluted bacterial suspension was taken from the tubes, evenly spread onto the MHA plate, and incubated for 18 h at 37 °C. The plate was removed, and photographed, recording the number of colonies.

## Results and discussion

### Structural and chemical characteristics

#### Phase structure and grain size

**Figure 2:**
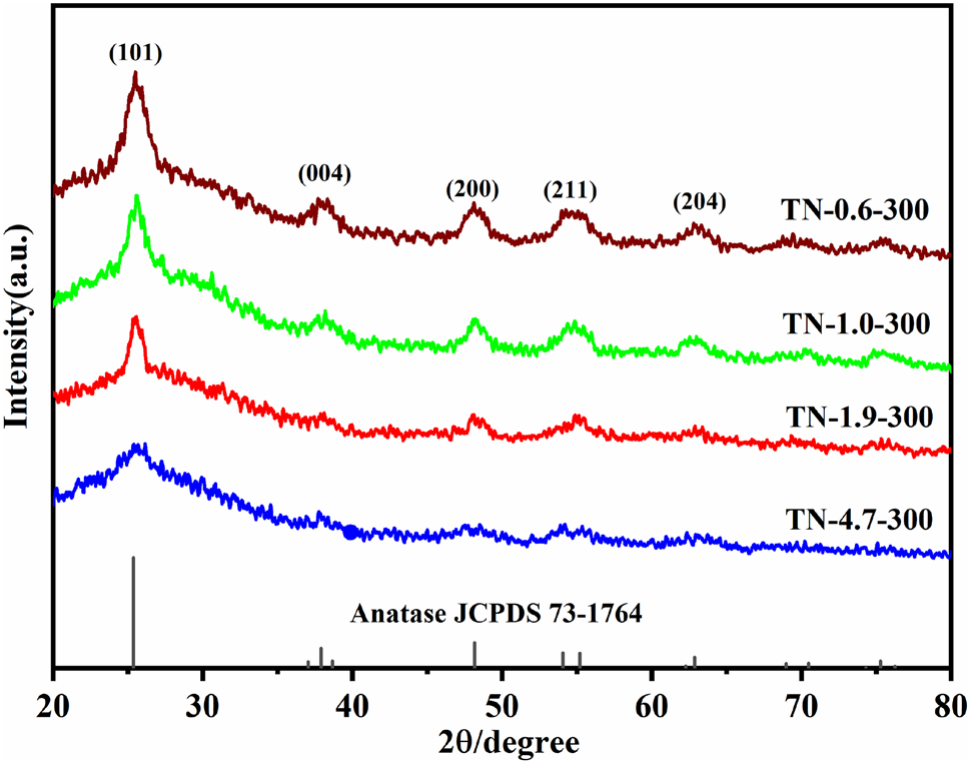
XRD patterns of TN-0.6-300, TN-1.0-300, TN-1.9-300 and TN-4.7-300.

The crystal configuration and grain size of TiO_2_ were analyzed using XRD for sample preparation at different pH and calcination temperature. The characteristic diffraction peaks at 2θ of 25.37°, 37.91°, 48.16°, 51.05°, 55.20°, and 62.87° correspond to the (101), (004), (200), (105), (211), and (204) planes of crystalline anatase (JCPDS 73-1764), respectively. The TiO_2_ samples prepared under all conditions exhibited the anatase phase (Fig. S1). A conversion of TiO_2_ from anatase to rutile was observed in the case of TN-0.6-700. At a high concentration of HCl, the surface energy of the TiO_2_ crystals increases, which facilitates conversion of anatase to rutile^33^. The crystallinity of TiO_2_ was reduced at a calcination temperature of 300 °C and deteriorated with increasing pH (Fig. 2). The findings suggest that a decrease in pH is accompanied by larger crystalline grains. A positive correlation between calcination temperature and TiO_2_ grain size, as determined by the Scherrer equation, is shown in Fig. S2. It should be noted that sample TN-4.7, which did not undergo hydrochloric acid treatment, exhibited the largest grain size. A slight decrease in grain size was observed with decreasing pH after the addition of hydrochloric acid. The grain sizes of TN-0.6-300, TN-1.0-300, TN-1.9-300, and TN-4.7-300 were 8.7, 9.9, 11.4, and 16.1 nm, respectively. The observed dependence of grain size on pH may be attributed to the repulsive effect of H^+^ with respect to positively charged Ti ions when HCl is added, resulting in the formation of TiO_2_ with a smaller grain size^34^.

#### TEM analysis

**Figure 3:**
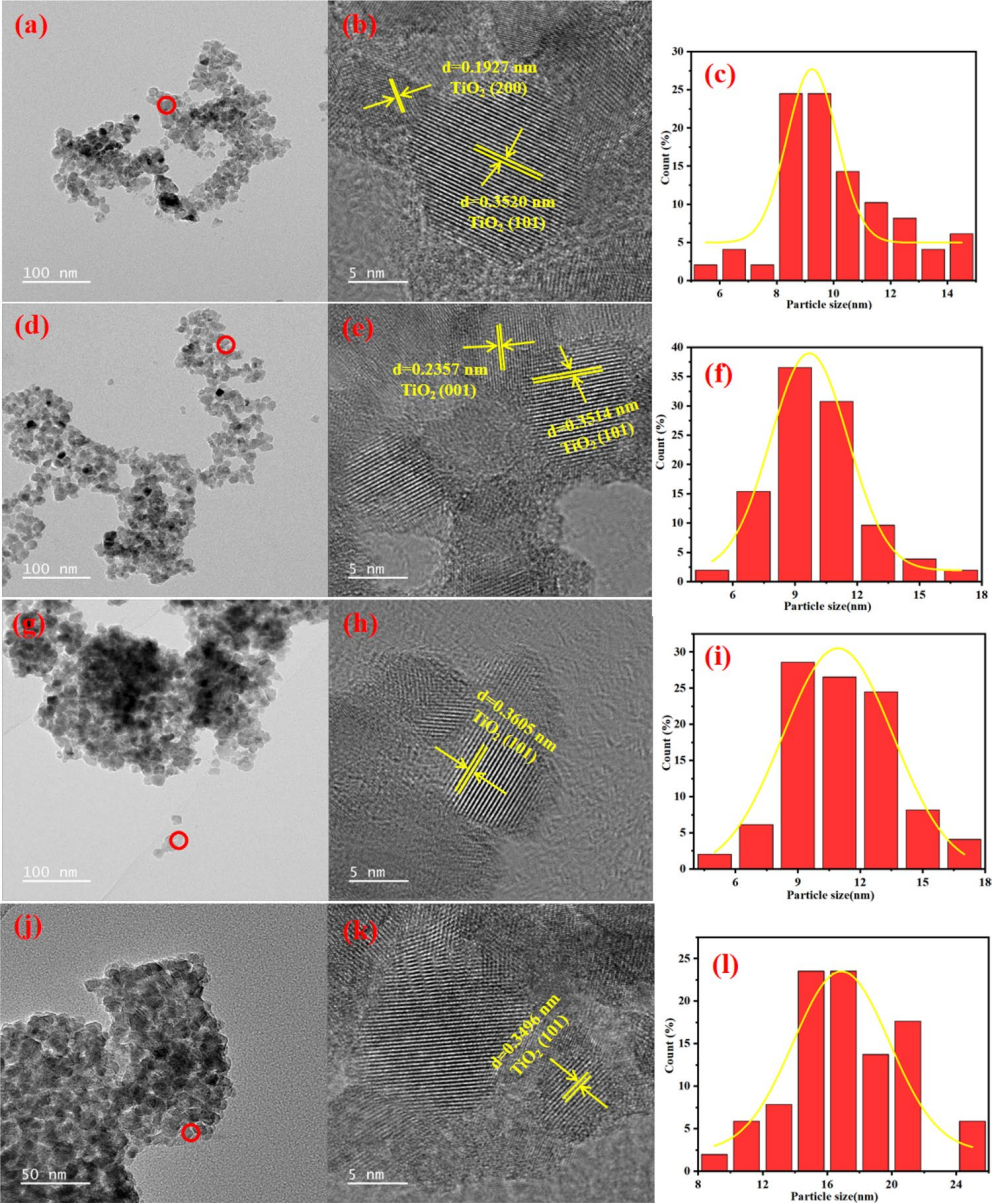
TEM, HRTEM and particle size distribution of TiO_2_ prepared at different pH: (**a**, **b**, **c**: TN-0.6-300); (**d**, **e**, **f**: TN-1.0-300); (**g**, **h**, **i**: TN-1.9-300); (**j**, **k**, **l**: TN-4.7-300).

The surface morphology, grain size, and exposed crystal surface of TN-0.6-300, TN-1.0-300, TN-1.9-300, and TN-4.7-300 were analyzed by transmission electron microscopy (TEM) and high-resolution transmission electron microscopy (HRTEM), as shown in Fig. 3. The TEM images offer evidence of sample spherical morphology with well-defined grain boundaries. The results indicate that pH does not significantly affect the morphology of TiO_2_. The HRTEM images reveal that the primary lattice spacing for TiO_2_ prepared at different pH is ca. 0.35 nm. This spacing corresponds to the (101) anatase plane, which is known for exhibiting superior thermal stability relative to other facets of anatase^35^. It should be noted that the HRTEM analysis of TN-0.6-300 has revealed the presence of the (101) plane associated with the exposed crystal surface, and the (200) plane with a lattice spacing of ca. 0.19 nm (Fig. 3b). In contrast, TN-1.0-300 exhibits the (001) plane with a lattice spacing of ca. 0.24 nm, as illustrated in Fig. 3e. The histograms illustrating particle size distributions have established average particle sizes for TN-0.6-300, TN-1.0-300, TN-1.9-300, and TN-4.7-300 of 10.1, 9.9, 11.2, and 17.0 nm, respectively. The observed grain sizes at the four pH values are consistent with the grain sizes calculated using the Scherrer equation.

#### XPS and EPR analysis

**Figure 4:**
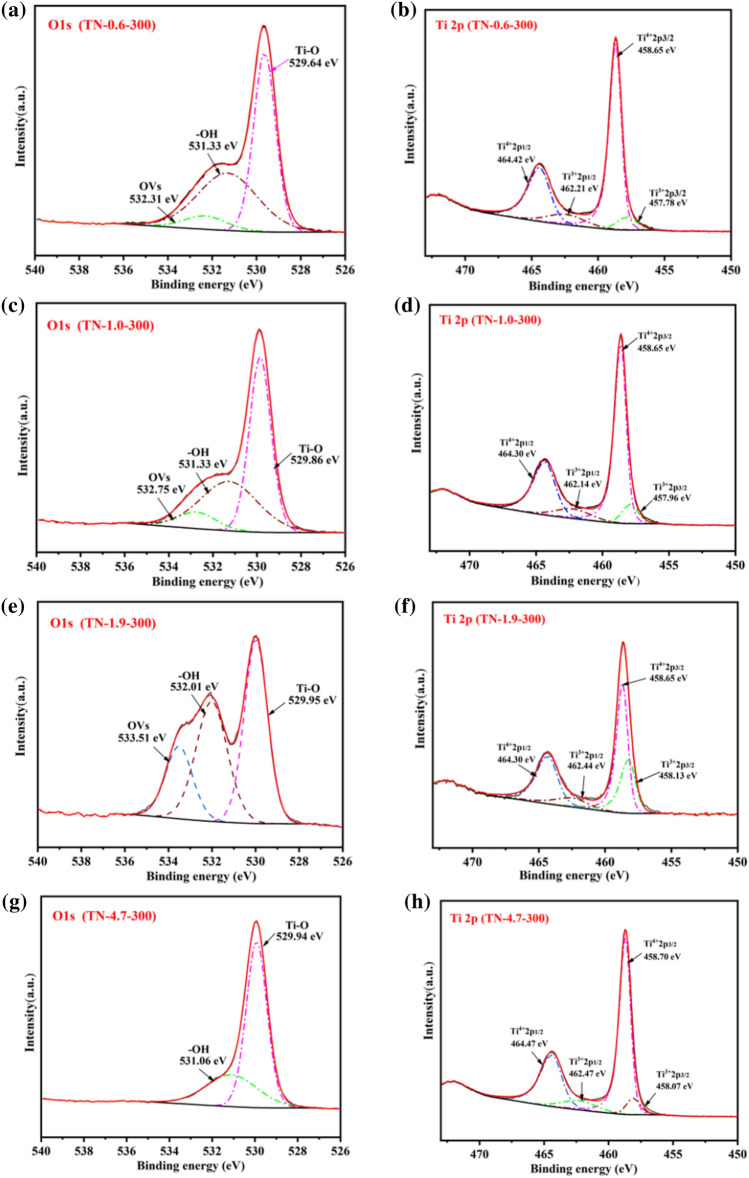
XPS high-resolution O1s and Ti2p spectra for TiO_2_ prepared at different pH: (**a**, **b**: TN-0.6-300); (**c**, **d**: TN-1.0-300); (**e**, **f**: TN-1.9-300); (**g**, **h**: TN-4.7-300).

XPS measurements were employed to analyze the surface chemical composition and valence states of the samples. The high-resolution O1s and Ti2p spectra for TN-0.6-300, TN-1.0-300, TN-1.9-300, and TN-4.7-300 are presented in Fig. 4a–h. It can be seen that the O1s spectrum presents three peaks with binding energies of 529.6, 531.3, and 532.4 eV. These peaks are associated with Ti–O–Ti bonds, and surface adsorbed –OH, and OVs that are in close proximity to Ti^3+^, respectively, which provide evidence for the presence of Ti^3+^^36–38^. The Ti 2p spectrum exhibits four characteristic peaks at binding energies of 458.65, 464.42, 457.78, and 462.21 eV. These peaks can be attributed to the 2p_3/2_ and 2p_1/2_ orbitals of Ti^4+^ and Ti^3+^ ions, respectively, confirming the presence of Ti^3+^. The intensity of Ti^3+^ followed the order, TN-1.9-300 > TN-1.0-300 > TN-0.6-300 > TN-4.7-300, with values of 10.69%, 9.87%, 9.32%, and 8.86%, respectively. It should be noted that the –OH and OVs peaks for TN-1.9-300 are shifted by 0.76 and 0.68 eV to higher binding energies, respectively. This shift can be attributed to a lattice distortion caused by a strong interaction between OVs and Ti^4+^ ions. High concentrations of OVs, which maintain a positive charge, serve to repel Ti towards neighboring OVs. This repulsion reduces the Ti–O bond length and increases the binding energy of O1s^39^.

**Figure 5:**
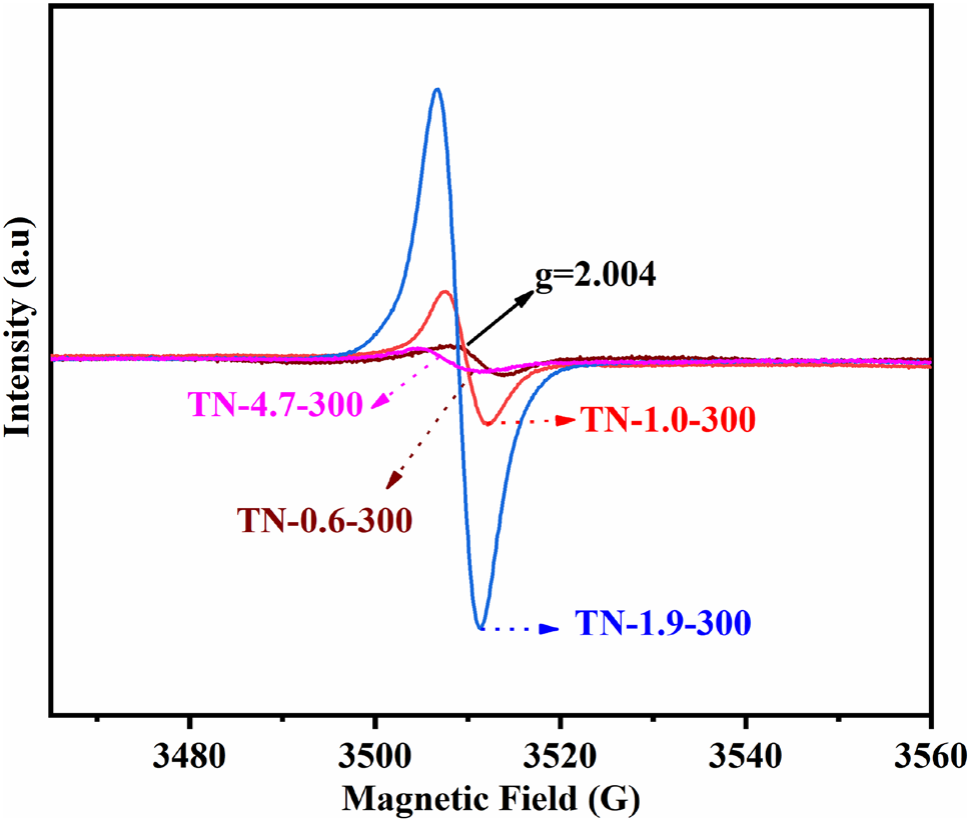
EPR spectrum of OVs associated with TN-0.6-300, TN-1.0-300, TN-1.9-300, and TN-4.7-300.

The presence of OVs is further confirmed by the EPR analysis, shown in Fig. 5. The EPR spectra of as-prepared samples show intense axially symmetry signals centered on the g = 2.004, which have been extensively reported for Ti^3+^ ions in anatase crystallite^40,41^. Moreover, the variation in the EPR signal associated with OVs is consistent with the order of Ti^3+^ content determined by XPS. This means that the intensity of OVs decreased as the pH decreased in the presence of HCl as a catalyst. At low pH, the TBOT hydrolysis rate is inhibited, resulting in incomplete hydrolysis, with a consequent generation of OVs^42^. Further investigation is required to elucidate the underlying mechanism whereby pH influences the production of OVs. It is obvious that the signal intensities associated with OVs is consistent with the order of Ti^3+^ content determined by XPS. The presence of OVs decreases the rate of TiO_2_ photogenerated e^−^–h^+^ recombination, increasing the rate of reactive oxygen species (ROS) generation, and enhances the generation of hydroxyl groups on the surface of TiO_2_, thereby contributing to photocatalytic activity^43,44^.

#### BET surface areas and pore distributions

**Figure 6:**
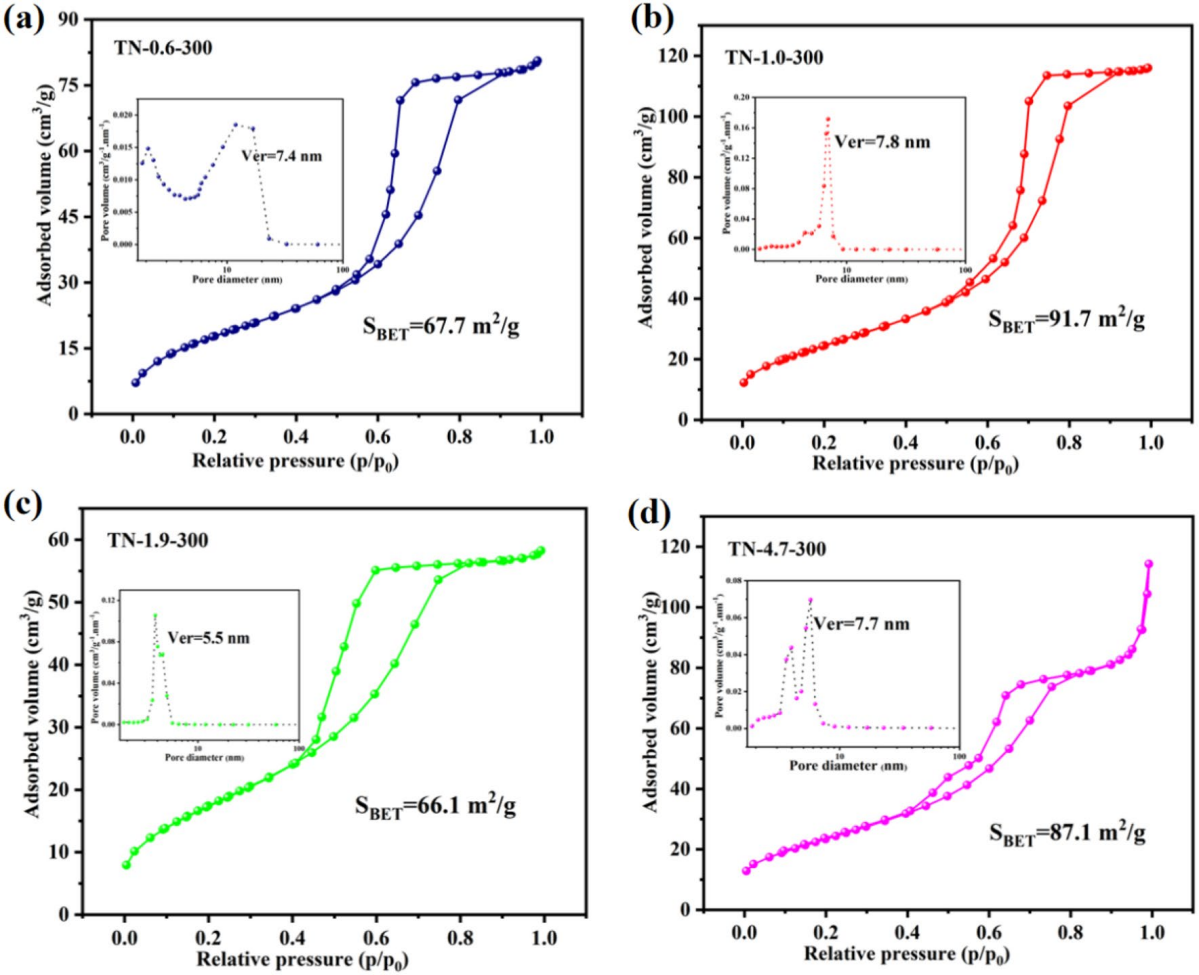
Nitrogen adsorption–desorption isotherms and corresponding pore size distributions (inset) for (**a**) TN-0.6-300, (**b**) TN-1.0-300, (**c**) TN-1.9-300, and (**d**) TN-4.7-300.

The specific surface area and pore size distribution of TiO_2_ samples prepared at different pH were determined from low-temperature nitrogen adsorption–desorption measurements, and the results are shown in Fig. 6. The isotherms of all the samples correspond to type IV (BDDT classification), exhibiting an obvious hysteresis loop (Type H2), indicating a mesoporous structure^45^. Both the specific surface area and pore size decreased in the order, TN-1.0-300 > TN-4.7-300 > TN-0.6-300 > TN-1.9-300. The corresponding surface areas are 91.7, 87.1, 67.7, and 66.1 m^2^/g, and the associated pore sizes are 7.8, 7.7, 7.4, and 5.5 nm, respectively. The TN-1.0-300 sample exhibited a narrow pore size distribution (Fig. 6b), indicating the formation of regular pore channels in the mesoporous region. Moreover, TN-1.0-300 is characterized by a higher specific surface area and larger grain size, which should result in enhanced photocatalytic activity. A high surface area increases the number of available active sites for the adsorption of pollutant molecules and reduces the rate of electron–hole complexation^46^. This, in turn, enhances the efficiency of photocatalytic degradation of dyes. In addition, a larger average pore size facilitates the entry of organic pollutants into the pores, thereby increasing the efficiency of dye decomposition^47^.

#### Optical properties

**Figure 7:**
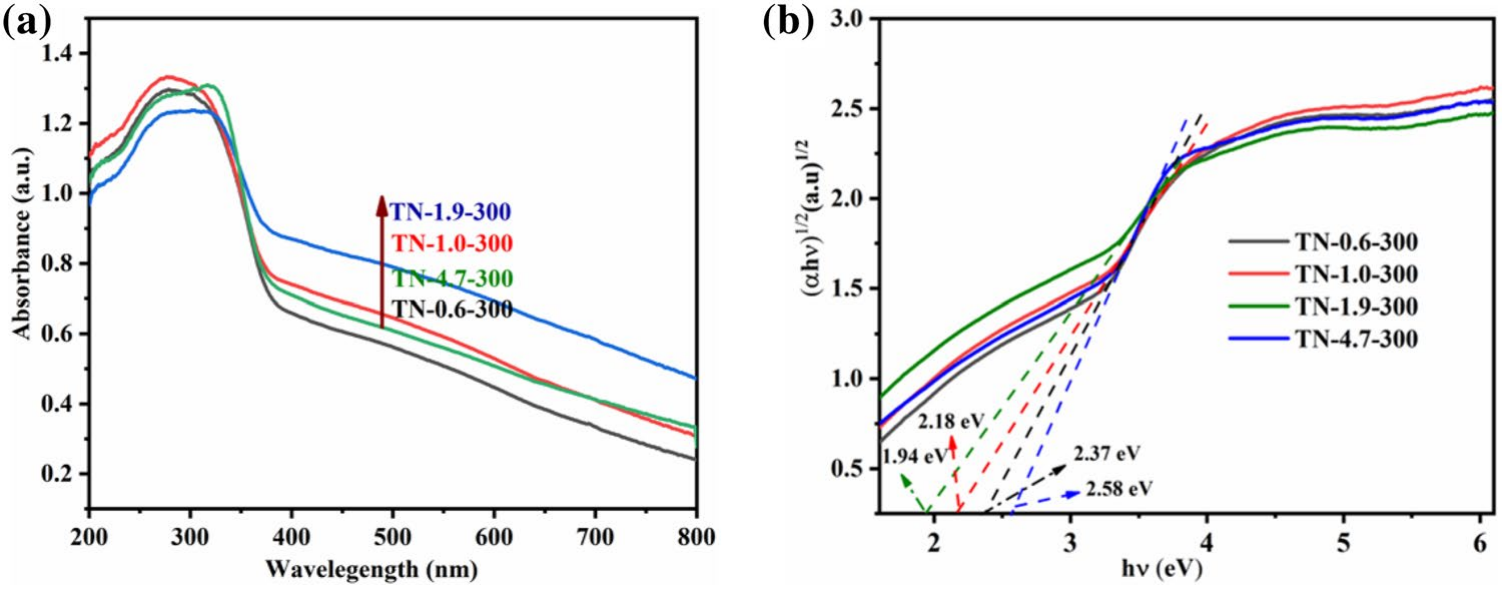
(**a**) UV–Visible diffuse reflectance spectra, and (**b**) Kubelka–Munk function versus photon energy for TN-0.6-300, TN-1.0-300, TN-1.9-300, and TN-4.7-300.

The spectral absorption range and energy band gap of TiO_2_ were examined using UV–Vis DRS to identify samples that demonstrate a high level of light absorption, a narrow energy band gap, and a shift to longer wavelengths in terms of light absorption. Based on the UV–Vis absorption spectra presented in Fig. 7a and Fig. S3a, it is evident that all the samples show a significant optical absorption peak below 400 nm. This peak can be attributed to the absorption associated with the intrinsic band gap of TiO_2_. In the case of TN-1.0-300, the optical absorption edge of TiO_2_ is slightly red-shifted to ca. 410 nm when the calcination temperature was increased from 400 to 700 °C. At a calcination temperature of 300 °C, TiO_2_ prepared at different pH exhibited strong light absorption in the visible wavelength range from 400 to 800 nm. The dependence of the energy band gap of TiO_2_ on calcination temperature is presented in Fig. S3c, where it is evident that sample synthesis at 300 °C resulted in the smallest energy band gap. The band gaps, as shown in Fig. 7b, follow the order, TN-4.7-300 > TN-0.6-300 > TN-1.0-300 > TN-1.9-300, with corresponding values of 2.58, 2.37, 2.18, and 1.94 eV, respectively, which are all lower than 3.2 eV^21^. The relationship between the energy band gap and the intensity of the EPR OVs signal indicates that a smaller energy band gap is associated with a stronger signal. This response suggests that the presence of Ti^3+^ and OVs is an important factor contributing to the redshift in the optical absorption range and the decrease in the energy band gap of TiO_2_^48^. In addition, the enhanced absorbance in the visible range of the samples may be related to the presence of impurities caused by incomplete decomposition of organic compound and amorphous metatitanic acid (Ti(OH)_4_) due to low calcination temperature (Figs. S4, S5).

### Self-cleaning properties of TiO_2_

#### Photocatalytic degradation activity

The photocatalytic activity of TiO_2_, prepared under different conditions, was assessed by measuring the degradation rate of MO solutions under simulated sunlight and visible light radiation. Before subjecting the MO to radiation degradation using a xenon lamp as a light source, all the samples reached adsorption–desorption equilibrium at 700 rpm in the dark. Blank experiments established that the concentration of MO did not change in the absence of a TiO_2_ catalyst.

**Figure 8:**
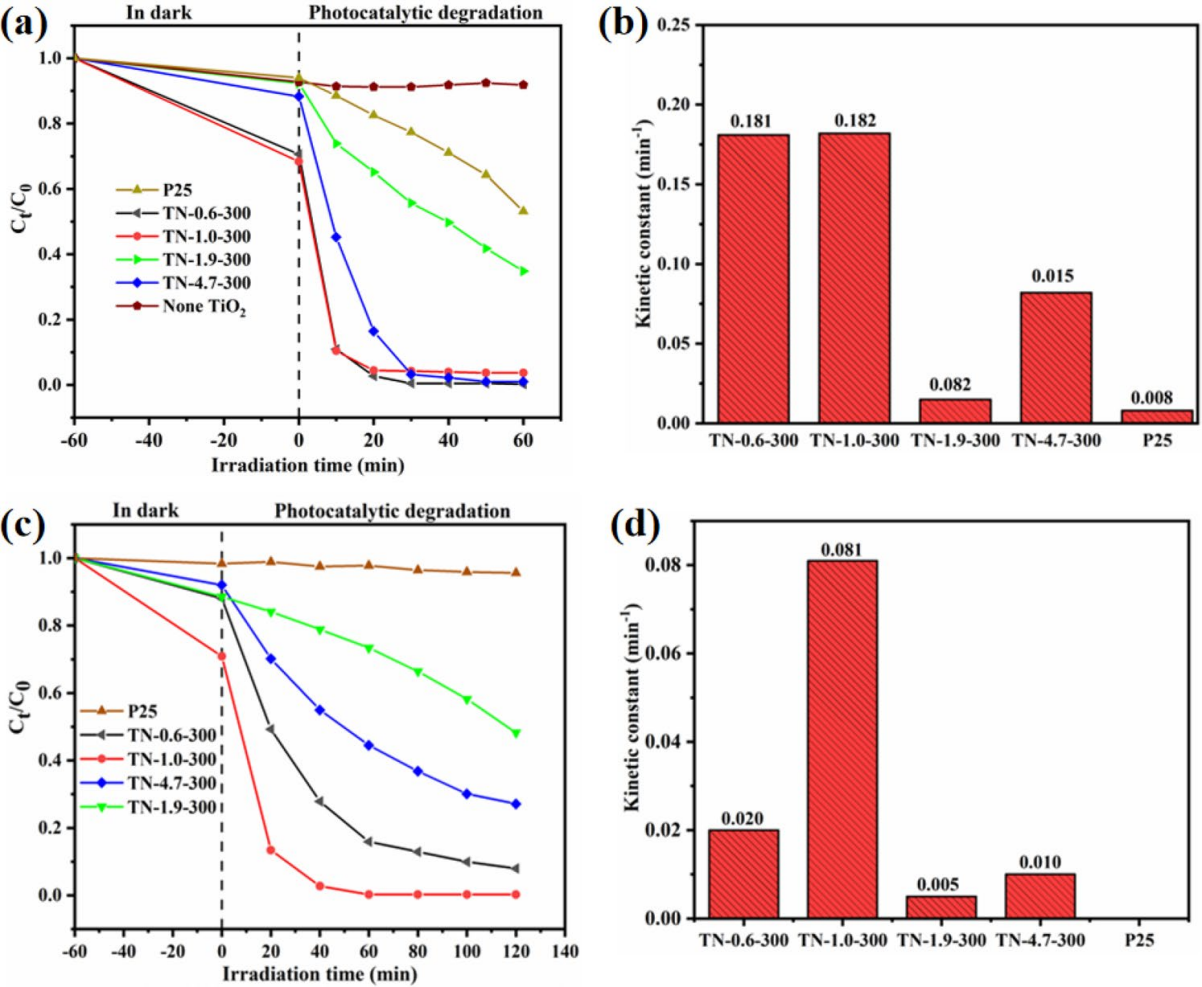
(**a**,**b**) Photocatalytic activities under full spectrum of as-prepared samples; (**c**,**d**) Photocatalytic activities under visible light of as-prepared samples.

In order to investigate the impact of calcination temperature (300–700 °C) on the photocatalytic degradation of MO, TN-1.0 was chosen as a representative system. The results, presented in Fig. S6, indicate that MO photocatalytic degradation efficiency decreased with increasing calcination temperature under both simulated sunlight (Fig. S6a) and visible light irradiation (Fig. S6b). This can be attributed to the increase in grain size at higher temperatures established by XRD analysis, where a larger particle size lowers the number of active sites^49^. It was observed that TiO_2_ delivered lower photocatalytic activity under visible light at temperatures above 400 °C, but the activity at 300 °C was only slightly lower than that achieved under simulated sunlight. This can be ascribed to the lower energy band gap of TiO_2_ at this temperature. The MO degradation curves under simulated sunlight for samples calcined at 300 °C with varying pH are shown in Fig. 8a. The photocatalytic activity decreased in the order, TN-1.0-300 ≥ TN-0.6-300 > TN-4.7-300 > TN-1.9-300 > P25. The associated first-order kinetic constants (k) exhibit the same trend, with values of 0.182, 0.181, 0.082, 0.015 and 0.008 min^−1^, respectively. (Fig. 8b). A switch to visible light radiation resulted in an increase in reaction time from 60 to 120 min (Fig. 8c). After 120 min of visible light irradiation, MO concentration did not change over TiO_2_ (P25), but the as-prepared samples exhibited good visible light activity. The trends in terms of photocatalytic activity response to pH levels were consistent with that observed for simulated solar radiation. The TN-1.0-300 catalyst delivered the highest level of MO photodegradation, followed by TN-0.6-300 where the conversion was significantly lower. The variation of the rate constant (Fig. 8d) follows the same trend as observed under simulated solar radiation, with values of 0.081, 0.020, 0.010 and 0.005 min^−1^ for TN-1.0-300, TN-0.6-300, TN-4.7-300 and TN-1.9-300, respectively.

**Figure 9:**
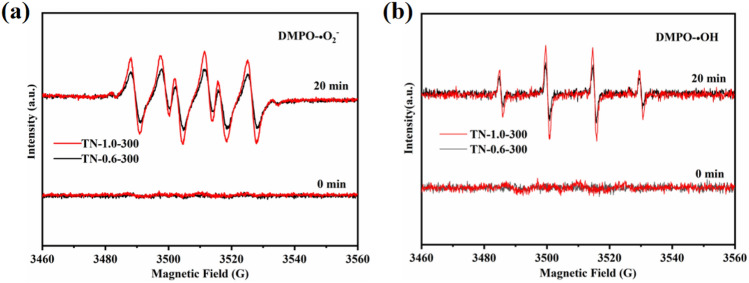
EPR spectra of (**a**) DMPO-**·**O_2_^−^, and (**b**) DMPO-**·**OH for TN-0.6-300 and TN-1.0-300.

The enhanced photocatalytic activity associated with TN-1.0-300 under both simulated sunlight and visible light can be attributed to several factors. Firstly, the presence of Ti^3+^ and OVs reduces the energy band gap of TiO_2_ and elevates photocatalytic activity. The photocatalytic activity of TiO_2_ is directly proportional to the content of Ti^3+^ and OVs. However, an excessive Ti^3+^ and OVs content in TN-1.9-300 results in the formation of an e^−^–h^+^ complex center that lowers photocatalytic activity^50^. Photogenerated radical production, as tested by EPR, has revealed that the characteristic signal peaks due to DMPO-**·**O_2_^−^ and DMPO-**·**OH appeared in the spectra for both TN-1.0-300 and TN-0.6-300 after 20 min of radiation under visible light. Moreover, the production rates of **·**O_2_^−^ and **·**OH in TN-1.0-300 were faster than TN-0.6-300, as shown in Fig. 9. This suggests that TN-1.0-300 exhibits a lower photogenerated e–h^+^ complexation rate than TN-0.6-300, and generates more free radicals under the same conditions. The appearance of OVs, **·**O_2_^−^, and **·**OH confirms a high photocatalytic activity for TN-1.0-300. In addition, TiO_2_ prepared under these conditions is characterized by a smaller grain size and higher crystallinity. A decrease in grain size enhances the diffusion rate of photogenerated carriers from the interior of TiO_2_ to the surface, resulting in an increased photocatalytic efficiency^51^. TN-1.0-300 also exhibits the largest specific surface area and pore size, which facilitates light absorption due to the greater number of active sites^52^. Furthermore, the presence of exposed (001) crystalline planes, established by TEM analysis, contributes to the improved photocatalytic performance of TN-1.0-300. Photogenerated electrons are mainly enriched in the (101) plane, whereas photogenerated holes predominate in the (001) plane facets. The occurrence of both (001) and (101) crystalline planes in a certain ratio forms crystalline heterojunctions, which serve to reduce the recombination of electrons and holes^53^.

#### Hydrophilic properties

**Figure 10:**
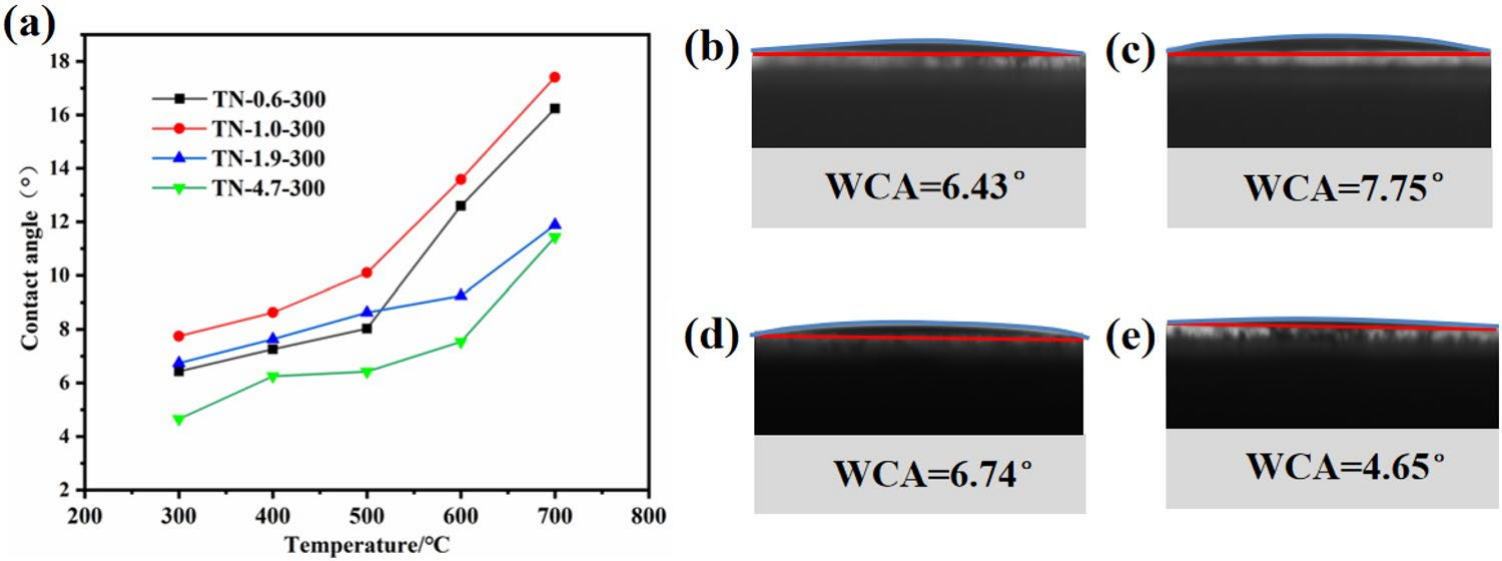
(**a**) Variation of TiO_2_ water contact angle with different pH and temperature. Water contact angles of (**b**) TN-0.6-300, (**c**) TN-1.0-300, (**d**) TN-1.9-300 and (**e**) TN-4.7-300.

The hydrophilicity of TiO_2_ prepared using different process conditions was assessed by observing the water contact angle (WCA) of droplets on a film under natural light conditions, as shown in Fig. 10a. It can be seen that the WCA ranges from 4.6° to 17.4°, indicating that the TiO_2_ samples exhibit excellent hydrophilic properties, and can even reach the superhydrophilic level. The WCA increased with increasing calcination temperature for different pH values. Given the relationship of grain size with temperature, it can be inferred that changes in grain size are positively correlated with the contact angle. This observation offers new possibilities for enhancing material hydrophilicity by regulating morphology. However, TN-4.7-300 which has the largest grain size was characterized by the smallest WCA. This indicates that the factors affecting the hydrophilicity of TiO_2_ are not singular^54^.

The contact angle of TiO_2_, prepared at various pH values and calcined at 300 °C, has been measured and the results are presented in Fig. 10b–e. Sample TN-1.0-300 exhibited the largest WCA, followed by TN-1.9-300, TN-0.6-300, and the smallest WCA was associated with TN-4.7-300; the values are 7.8, 6.7, 6.4, and 4.7°, respectively. Based on the findings of Yang et al.^55^, who controlled the synthesis of hydrophilic or hydrophobic membranes by manipulating the hydrolysis-condensation rate of silica precursors, it can be inferred that the variation in TiO_2_ WCA at different pH may be attributed to the extent of TBOT hydrolysis caused by the addition of varying amounts of HCl. An increased HCl addition, with a consequent lower pH, inhibited hydrolysis to a greater extent. However, TBOT can be synthesized in the form of a titanium sol that consists of hydrophilic (–OH) and hydrophobic (–CH_3_) groups due to complete and incomplete hydrolysis, respectively. At a pH of 4.7, the TBOT precursor underwent complete hydrolysis without the addition of HCl as a hydrolysis inhibitor, leading to the optimal hydrophilic characteristics observed in TN-4.7-300. Moreover, TN-1.9-300 and TN-1.0-300 exhibited hydrophilic properties that are consistent with this reasoning. However, this is not the case for TN-0.6-300, which exhibits high hydrophilicity due to the high hydroxyl content, as determined by XPS analysis^56^. The results have established that TiO_2_ exhibits the best hydrophilic properties at a calcination temperature of 300 °C.

#### Inhibition of bacterial adhesion

**Figure 11:**
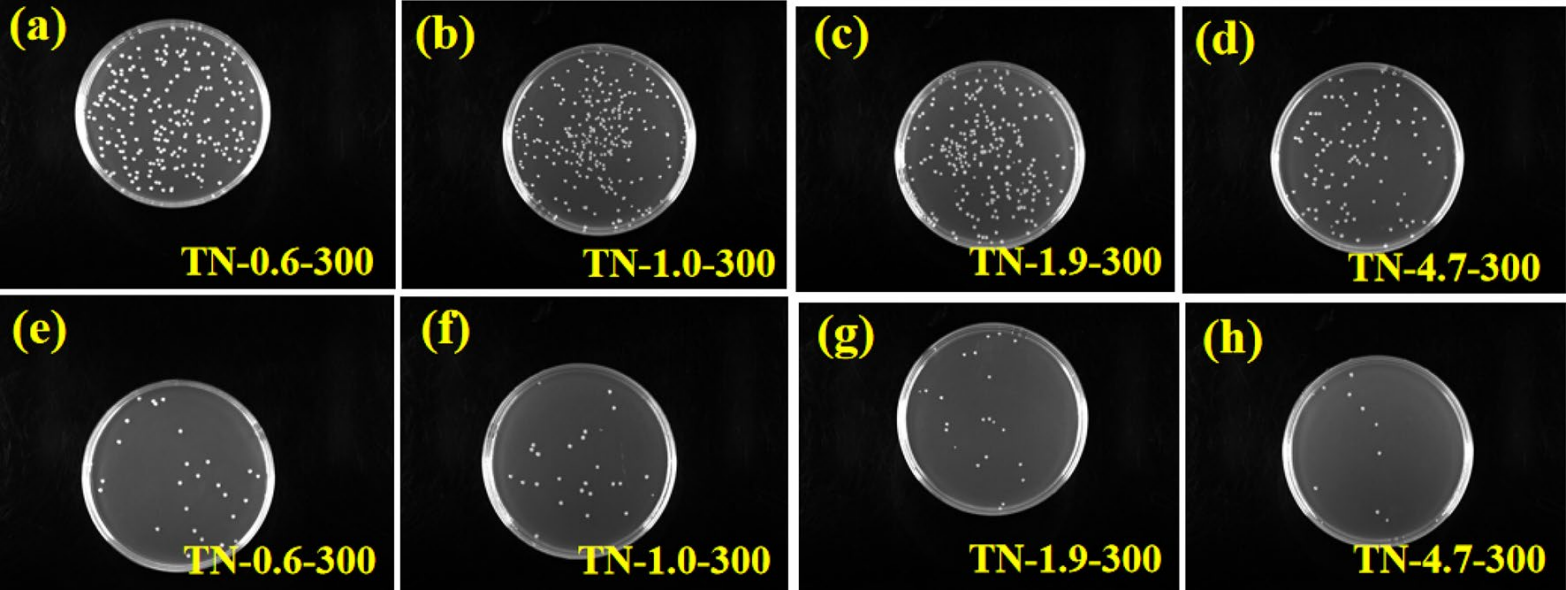
Images of E. coli colonies on TiO_2_ films (**a**, **e**: TN-0.6-300); (**b**, **f**: TN-1.0-300); (**c**, **g**: TN-1.9-300); (**d**, **h**: TN-4.7-300). Note: The dilution associated with images (**a**–**d**) was 1 × 10^2^, and 1 × 10^3^ for images (**e**–**h**).

The adhesion E. coli to TiO_2_ films is illustrated in Fig. 11a–d for bacteria diluted by a factor of 1 × 10^2^ before incubation, and in Fig. 11e–h for bacteria diluted by a factor of 1 × 10^3^. Following bacteria adherence to the surface of different films, and subsequent dilution and incubation using the plate spread method, the number of bacteria colonies on the films decreased in the order, TN-1.0-300 > TN-1.9-300 > TN-0.6-300 > TN-4.7-300. The corresponding surface bacterial adherence was 4.26 × 10^6^ CFU, 3.90 × 10^6^ CFU, 3.86 × 10^6^ CFU, and 1.90 × 10^6^ CFU, respectively. The concentration of bacterial solution in PBS was 2.13 × 10^5^ CFU/mL, 1.95 × 10^5^ CFU/mL, 1.93 × 10^5^ CFU/mL, and 9.50 × 10^4^ CFU/mL. The results suggest that TN-4.7-300 exhibited the best anti-bacterial adhesion response, followed by TN-0.6-300, while the performance of TN-1.9-300 was poor, and TN-1.0-300 showed a very limited resistance. Effective inhibition of bacterial adhesion may be attributed to a highly hydrophilic TiO_2_ surface^14^. The dependence of WCA on pH recorded in this study is consistent with the variation in resistance to bacterial adhesion, i.e., TN-1.0-300 > TN-1.9-300 > TN-0.6-300 > TN-4.7-300. This serves to confirm that an enhancement of TiO_2_ surface hydrophilicity can reduce bacterial adhesion. Bacteria spread and readily adhere to various materials and organisms, forming biofilms that are difficult to remove^57^. Therefore, it is crucial to develop highly hydrophilic surfaces that are resistant to bacterial adhesion.

#### Self-cleaning mechanism

**Figure 12:**
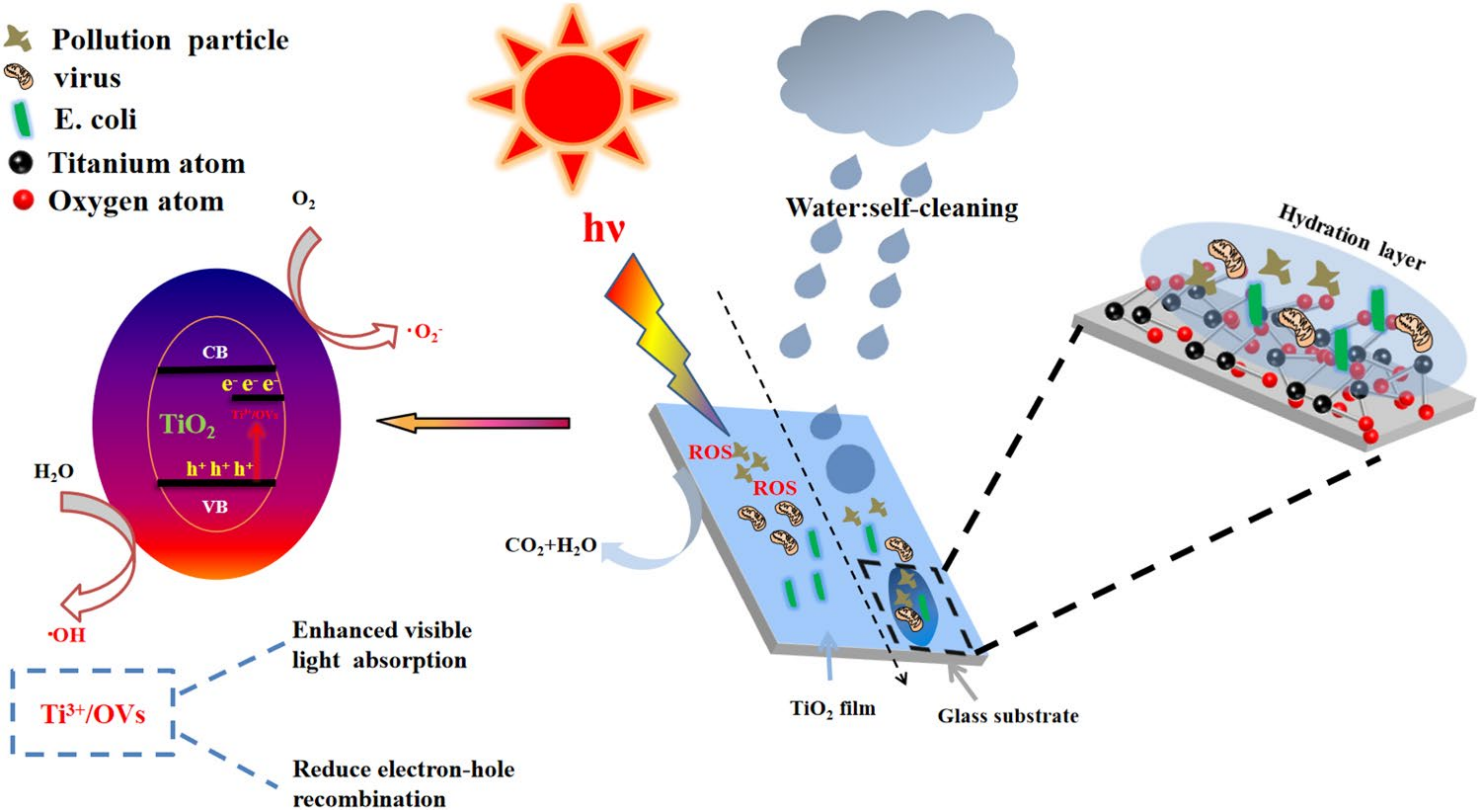
Schematic representation of the self-cleaning mechanism for TiO_2_ films.

Drawing on the above discussion, the self-cleaning mechanism of TiO_2_ is illustrated in Fig. 12. An incomplete hydrolysis of TBOT results in the generation of Ti^3+^ and OVs defects, which can reduce the energy band gap in TiO_2_ and enhance the response to visible light. The formation of Ti^3+^ and OVs can also facilitate movement of photogenerated electrons from the valence band (VB) to the Ti^3+^/OVs energy level. This process reduces the rate of complexation of photogenerated e^−^–h^+^ pairs. As a result, TiO_2_ generates **·**O_2_^-^ and **·**OH radicals when exposed to visible light, which enables the degradation of pollutants such as MO. In addition, a small TiO_2_ grain size enhances surface hydrophilic properties. When a water drop contacts the surface of ahydrophilic TiO_2_ film, a hydration layer is formed, which prevents adhesion of E. coli and circumvents bacterial contact infections. Ultimately, a self-cleaning TiO_2_ film with photocatalytic activity and resistance to bacterial adhesion under visible light radiation is produced.

## Conclusions

The crystal conformation and surface structure of anatase TiO_2_ can be controlled by regulating the hydrolytic condensation and thermal crystallization processes associated with the sol–gel method, with the successful introduction of Ti^3+^ and OV defects. The resultant materials exhibit good photocatalytic activity under visible light, and are hydrophilic and resistant to bacterial adhesion under natural light. Titania synthesis conditions, employing a pH of 1.0 and calcination temperature of 300 °C, yielded the most effective photodegradation catalyst under both simulated sunlight and visible light radiation. The associated first-order kinetic constants for the degradation of MO were 0.182 min^−1^ and 0.081 min^−1^, respectively. This is attributed to the large specific surface area of TN-1.0 where the (001) crystal plane is exposed. The occurrence of Ti^3+^ and OVs can reduce the band gap width of TiO_2_, which serves to enhance the photocatalytic effect under visible light. In addition, surface hydrophilicity can be controlled by adjusting the grain size. Under optimal conditions, the water contact angle of the TiO_2_ film under natural light was 7.75°, less than 10°, demonstrating excellent hydrophilic properties. The amount of E. coli bacteria adhering to TiO_2_ film decreased as the contact angle of the film surface decreased. This suggests that the hydrophilic TiO_2_ film forms a hydrated layer that repels E. coli and prevents bacterial adhesion.

### Supplementary Information

Supplementary Figures.

## Data Availability

All data generated or analysed during this study are included in this published article (and its Supplementary Information files).

## References

[1] Woolsey C (2022). Natural history of Sudan ebolavirus infection in rhesus and cynomolgus macaques. Emerg. Microbes Infect..

[2] Kumari R (2023). Antiviral approaches against influenza virus. Clin. Microbiol. Rev..

[3] Jackson CB, Farzan M, Chen B, Choe H (2022). Mechanisms of SARS-CoV-2 entry into cells. Nat. Rev. Mol. Cell Biol..

[4] Rodriguez-Morales AJ (2023). The global challenges of the long COVID-19 in adults and children. Travel Med. Infect. Dis..

[5] Msemburi W (2023). The WHO estimates of excess mortality associated with the COVID-19 pandemic. Nature.

[6] Imani SM (2020). Antimicrobial nanomaterials and coatings: Current mechanisms and future perspectives to control the spread of viruses including SARS-CoV-2. ACS nano.

[7] Arciola CR, Campoccia D, Montanaro L (2018). Implant infections: Adhesion, biofilm formation and immune evasion. Nat. Rev. Microbiol..

[8] Sungur, Ş. Titanium dioxide nanoparticles. *Handbook of Nanomaterials and Nanocomposites forEnergy and Environmental Applications*, 1–18 (2020).

[9] Zhu Z, Cai H, Sun D-W (2018). Titanium dioxide (TiO_2_) photocatalysis technology for nonthermal inactivation of microorganisms in foods. Trends Food Sci. Technol..

[10] Huang Y (2019). Protonated g-C_3_N_4_/Ti^3+^ self-doped TiO_2_ nanocomposite films: Room-temperature preparation, hydrophilicity, and application for photocatalytic NOx removal. Appl. Catal. B: Environ..

[11] Lai Y (2016). Recent advances in TiO2-based nanostructured surfaces with controllable wettability and adhesion. Small.

[12] Bergamonti L (2017). Enhanced self-cleaning properties of N-doped TiO_2_ coating for cultural heritage. Microchem. J..

[13] Padmanabhan NT, John H (2020). Titanium dioxide based self-cleaning smart surfaces: A short review. J. Environ. Chem. Eng..

[14] Madeira MD (2022). Depositation of sodium titanate nanotubes: Superhydrophilic surface and antibacterial approach. J. Mater. Res. Technol..

[15] Farkas B (2018). Optical, compositional and structural properties of pulsed laser deposited nitrogen-doped Titanium-dioxide. Appl. Surf. Sci..

[16] Prabhu S, Cindrella L, Kwon OJ, Mohanraju K (2017). Superhydrophilic and self-cleaning rGO-TiO_2_ composite coatings for indoor and outdoor photovoltaic applications. Solar Energy Mater. Solar Cells.

[17] Hosseini MS (2020). Investigation of the effective operational parameters of self-cleaning glass surface coating to improve methylene blue removal efficiency; application in solar cells. Solar Energy.

[18] Ratova M, Kelly PJ, West GT, Tosheva L, Edge M (2017). Reactive magnetron sputtering deposition of bismuth tungstate onto titania nanoparticles for enhancing visible light photocatalytic activity. Appl. Surf. Sci..

[19] Baker DR, Kamat PV (2009). Photosensitization of TiO_2_ nanostructures with CdS quantum dots: Particulate versus tubular support architectures. Adv. Funct. Mater..

[20] Li L (2020). Synthesis of Ti^3+^ self-doped mesoporous TiO_2_ cube with enhanced visible-light photoactivity by a simple reduction method. J. Alloys Compd..

[21] Gordon TR (2012). Nonaqueous synthesis of TiO_2_ nanocrystals using TiF_4_ to engineer morphology, oxygen vacancy concentration, and photocatalytic activity. J. Am. Chem. Soc..

[22] Prakash J, Cho J, Mishra YK (2022). Photocatalytic TiO_2_ nanomaterials as potential antimicrobial and antiviral agents: Scope against blocking the SARS-COV-2 spread. Micro Nano Eng..

[23] Yan Y (2019). Carbon quantum dot-decorated TiO_2_ for fast and sustainable antibacterial properties under visible-light. J. Alloys Compd..

[24] Kumaravel V (2021). Antimicrobial TiO_2_ nanocomposite coatings for surfaces, dental and orthopaedic implants. Chem. Eng. J..

[25] Wang R (2020). Graphdiyne-modified TiO_2_ nanofibers with osteoinductive and enhanced photocatalytic antibacterial activities to prevent implant infection. Nat. Commun..

[26] Hu J (2019). A new anti-biofilm strategy of enabling arbitrary surfaces of materials and devices with robust bacterial anti-adhesion via a spraying modified microsphere method. J. Mater. Chem. A.

[27] Jeon S, Lee J, Andrade J, De Gennes P (1991). Protein—surface interactions in the presence of polyethylene oxide: I. Simplified theory. J. Colloid Interface Sci..

[28] Wang S, Liu K, Yao X, Jiang L (2015). Bioinspired surfaces with superwettability: New insight on theory, design, and applications. Chem. Rev..

[29] Lu L, Zhu L, Liu X, Li J (2022). Self-cleaning mechanisms and laws of hydrophilic or hydrophobic surfaces of solar photovoltaic glass. Chem. Eng. Res. Design.

[30] Sethi SK, Manik G (2018). Recent progress in super hydrophobic/hydrophilic self-cleaning surfaces for various industrial applications: A review. Polym. Plast. Technol. Eng..

[31] Yohe ST, Colson YL, Grinstaff MW (2012). Superhydrophobic materials for tunable drug release: Using displacement of air to control delivery rates. J. Am. Chem. Soc..

[32] Grabowska E, Sobczak JW, Gazda M, Zaleska A (2012). Surface properties and visible light activity of W-TiO_2_ photocatalysts prepared by surface impregnation and sol–gel method. Appl. Catal. B: Environ..

[33] Chang SL, Ryu JH, Kim DH, Cho SY, Oh WC (2010). Reaction morphology and the effect of pH on the preparation of TiO_2_ nanoparticles by a sol–gel method. J. Ceram. Process. Res..

[34] Mustapha S (2021). Facile synthesis and characterization of TiO_2_ nanoparticles: X-ray peak profile analysis using Williamson-Hall and Debye-Scherrer methods. Int. Nano Lett..

[35] Selcuk S, Selloni A (2016). Facet-dependent trapping and dynamics of excess electrons at anatase TiO_2_ surfaces and aqueous interfaces. Nat. Mater..

[36] Pan J (2019). The enhancement of photocatalytic hydrogen production via Ti^3+^ self-doping black TiO_2_/g-C_3_N_4_ hollow core-shell nano-heterojunction. Appl. Catal. B: Environ..

[37] Wang P, Jia C, Li J, Yang P (2019). Ti^3+^-doped TiO_2_ (B)/anatase spheres prepared using thioglycolic acid towards super photocatalysis performance. J. Alloys Compd..

[38] Ni B, Jiang H, Guo WY, Xu Q, Min Y (2022). Tailoring the oxidation state of metallic TiO through Ti^3+^/Ti^2+^ regulation for photocatalytic conversion of CO_2_ to C_2_H_6_. Appl. Catal. B: Environ..

[39] Moya A (2015). Oxygen vacancies and interfaces enhancing photocatalytic hydrogen production in mesoporous CNT/TiO_2_ hybrids. Appl. Catal. B: Environ..

[40] Wang Y, Feng C, Zhang M, Yang J, Zhang Z (2010). Enhanced visible light photocatalytic activity of N-doped TiO_2_ in relation to single-electron-trapped oxygen vacancy and doped-nitrogen. Appl. Catal. B: Environ..

[41] Xia T, Zhang Y, Murowchick J, Chen X (2014). Vacuum-treated titanium dioxide nanocrystals: Optical properties, surface disorder, oxygen vacancy, and photocatalytic activities. Catal. Today.

[42] Karthik PV, Shaheer V, MahammedNeppolian AR, B.  (2019). Self-doping of Ti^3+^ in TiO_2_ through incomplete hydrolysis of titanium (IV) isopropoxide: An efficient visible light sonophotocatalyst for organic pollutants degradation. Appl. Catal. A. Gen. Int. J. Devoted Catal. Sci. Appl..

[43] Hao L, Miyazawa K, Yoshida H, Lu Y (2018). Visible-light-driven oxygen vacancies and Ti^3+^ co-doped TiO_2_ coatings prepared by mechanical coating and carbon reduction. Mater. Res. Bull..

[44] Sun Y (2018). Effect of heat treatment on surface hydrophilicity-retaining ability of titanium dioxide nanotubes. Appl. Surf. Sci..

[45] Hu M (2018). Ti^3+^ self-doped mesoporous black TiO_2_/SiO_2_/g-C_3_N_4_ sheets heterojunctions as remarkable visible-lightdriven photocatalysts. Appl. Catal. B: Environ..

[46] Alosfur FKM, Ridha NJ, Jumali MHH, Radiman S (2018). One-step formation of TiO_2_ hollow spheres via a facile microwave-assisted process for photocatalytic activity. Nanotechnology.

[47] Gerasimova T, Evdokimova O, Kraev A, Ivanov V, Agafonov A (2016). Micro-mesoporous anatase TiO_2_ nanorods with high specific surface area possessing enhanced adsorption ability and photocatalytic activity. Microporous Mesoporous Mater..

[48] Zhang C (2018). One-pot topotactic synthesis of Ti^3+^ self-doped 3D TiO_2_ hollow nanoboxes with enhanced visible light response. Chin. J. Catal..

[49] Wu N (2010). Shape-enhanced photocatalytic activity of single-crystalline anatase TiO_2_ (101) nanobelts. J. Am. Chem. Soc..

[50] Wang J (2009). Origin of photocatalytic activity of nitrogen-doped TiO_2_ nanobelts. J. Am. Chem. Soc..

[51] Meher S, Balakrishnan L (2014). Sol–gel derived nanocrystalline TiO_2_ thin films: A promising candidate for self-cleaning smart window applications. Mater. Sci. Semicond. Process..

[52] Zhou W (2011). Well-ordered large-pore mesoporous anatase TiO_2_ with remarkably high thermal stability and improved crystallinity: Preparation, characterization, and photocatalytic performance. Adv. Funct. Mater..

[53] Cao Y, Li Q, Li C, Li J, Yang J (2016). Surface heterojunction between (001) and (101) facets of ultrafine anatase TiO_2_ nanocrystals for highly efficient photoreduction CO_2_ to CH_4_. Appl. Catal. B: Environ..

[54] De Falco G (2018). TiO_2_ nanoparticle coatings with advanced antibacterial and hydrophilic properties prepared by flame aerosol synthesis and thermophoretic deposition. Surf. Coat. Technol..

[55] Yang X (2016). Controlled hydrophilic/hydrophobic property of silica films by manipulating the hydrolysis and condensation of tetraethoxysilane. Appl. Surf. Sci..

[56] Sun R, Chen Z, Peng J, Zheng T (2018). The effect mechanisms of pH, complexant and calcination temperature on the hydrophilicity of TiO_2_ films prepared by the sol-gel method. Appl. Surf. Sci..

[57] Garrett TR, Bhakoo M, Zhang Z (2008). Bacterial adhesion and biofilms on surfaces. Progr. Nat. Sci..

