# Supplementary material for: Visible light-induced photocatalytic and antibacterial adhesion properties of superhydrophilic TiO2 nanoparticles
Source: Sci Rep. 2024 Apr 4;14:7940. doi: 10.1038/s41598-024-58660-0 (PMC10995203; doi:10.1038/s41598-024-58660-0)
Supplement: Supplementary file 1 — Supplementary Figures. [file 41598_2024_58660_MOESM1_ESM.docx]

**Supporting Information**

**Visible light-induced photocatalytic and antibacterial adhesion properties of superhydrophilic TiO_2_ nanoparticles**

Mingzhu Zhou, Xingran Zhang, Yuanxia Quan, Yu Tian, Jie Cheng, Li Li^*^

School of Chemistry and Chemical Engineering, Chongqing University of Technology, Chongqing 400054, China

**
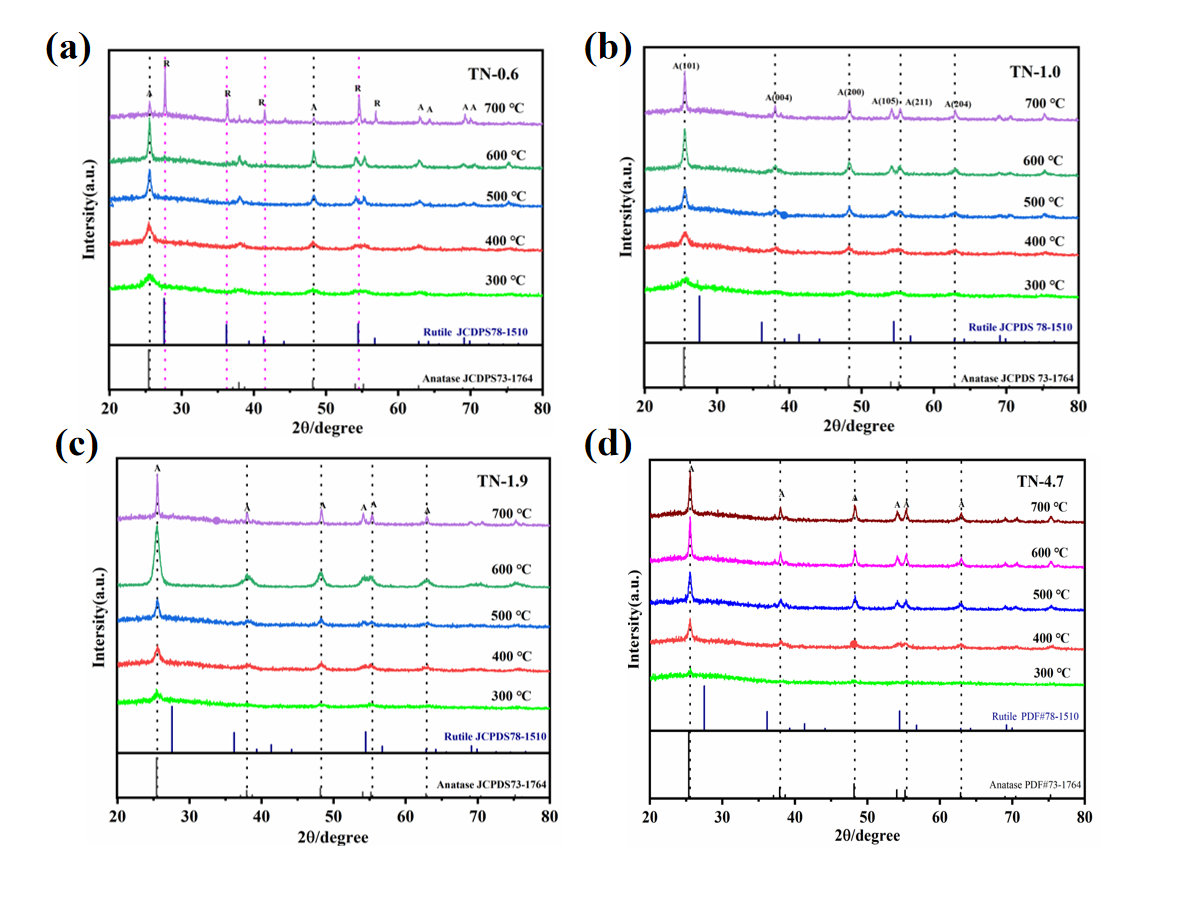
**

**Fig. S1.** XRD patterns of (a) TN-0.6 ,(b) TN-1.0 ,(b), TN-1.9 (c), and (d) TN-4.7 at different calcination temperatures.





**Fig. S2.** Variation of grain size of anatase TiO_2_ with pH and calcination temperature.

**
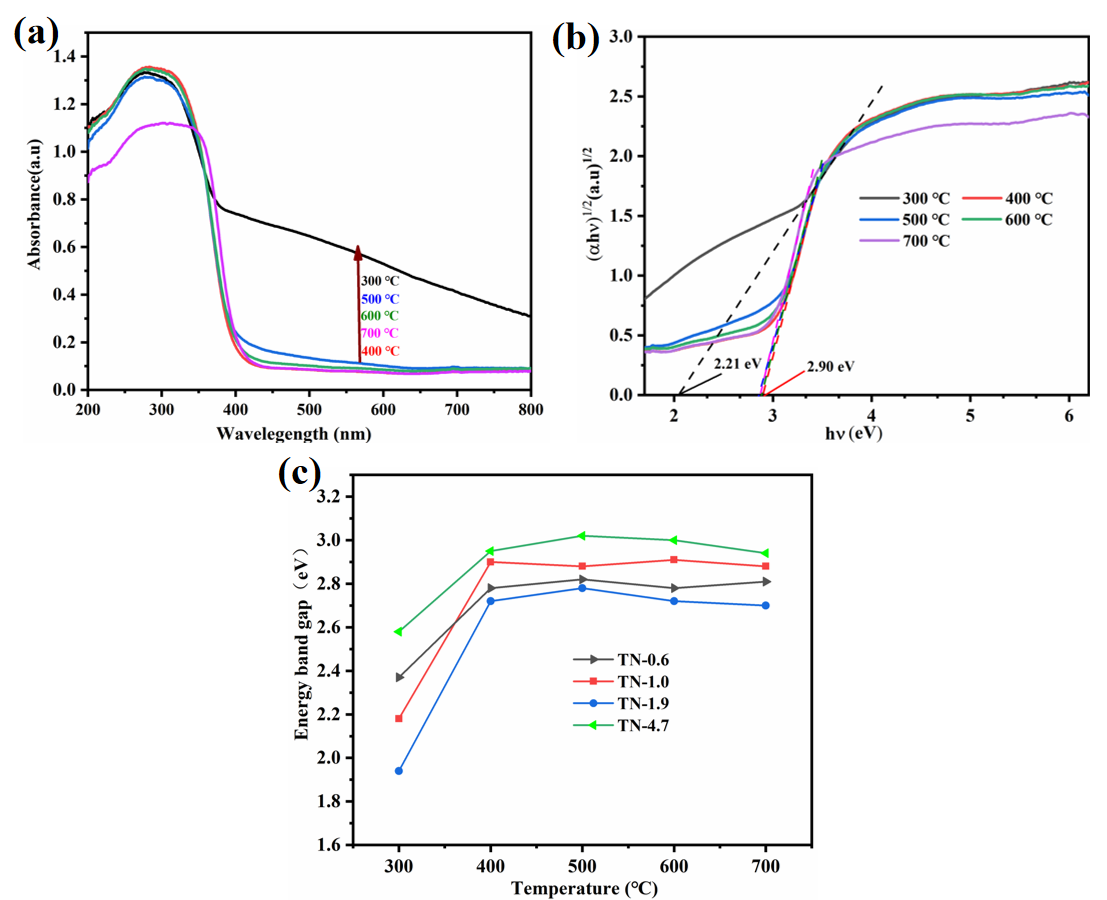
**

**Fig. S3.** (a) UV-Vis diffuse reflectance spectra and (b) the Kubelka-Munk function versus photon energy for TN-1.0 at different temperatures. (c) Variation of the energy band gap of TiO_2_ with different calcination temperatures and pH.


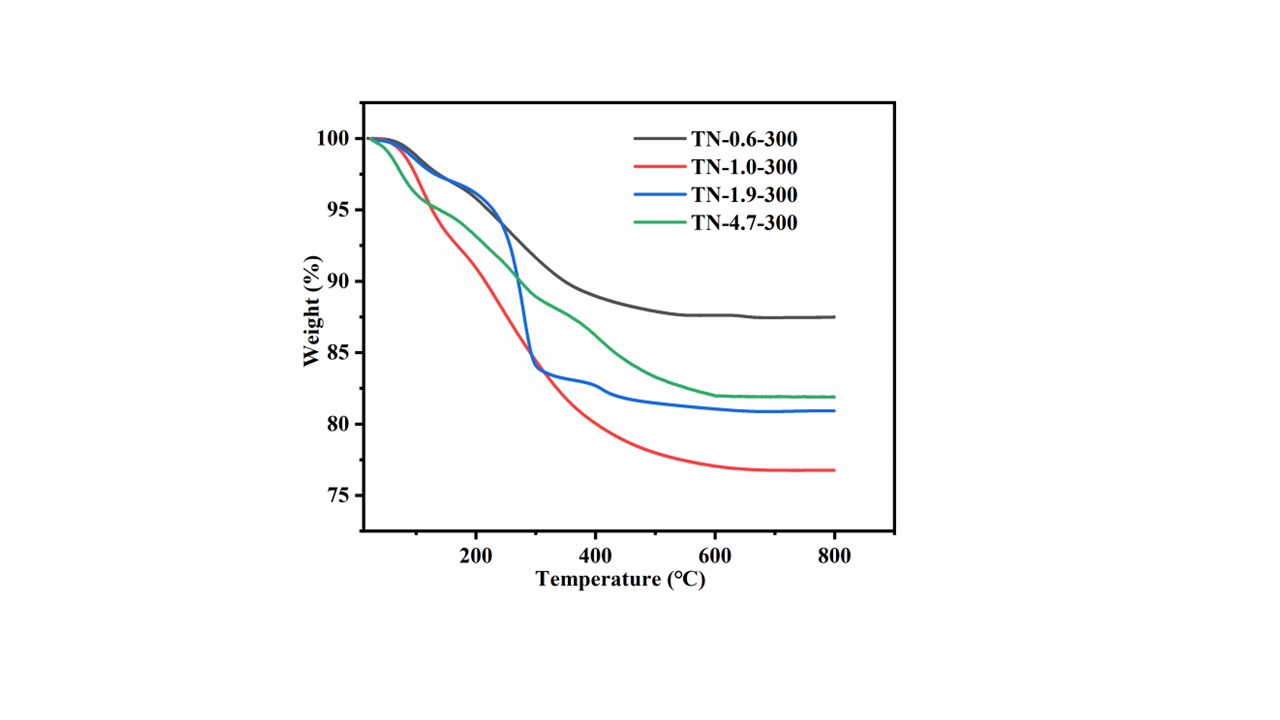


**Fig. S4.** Thermogravimetric analysis curve of the as-prepared samples.


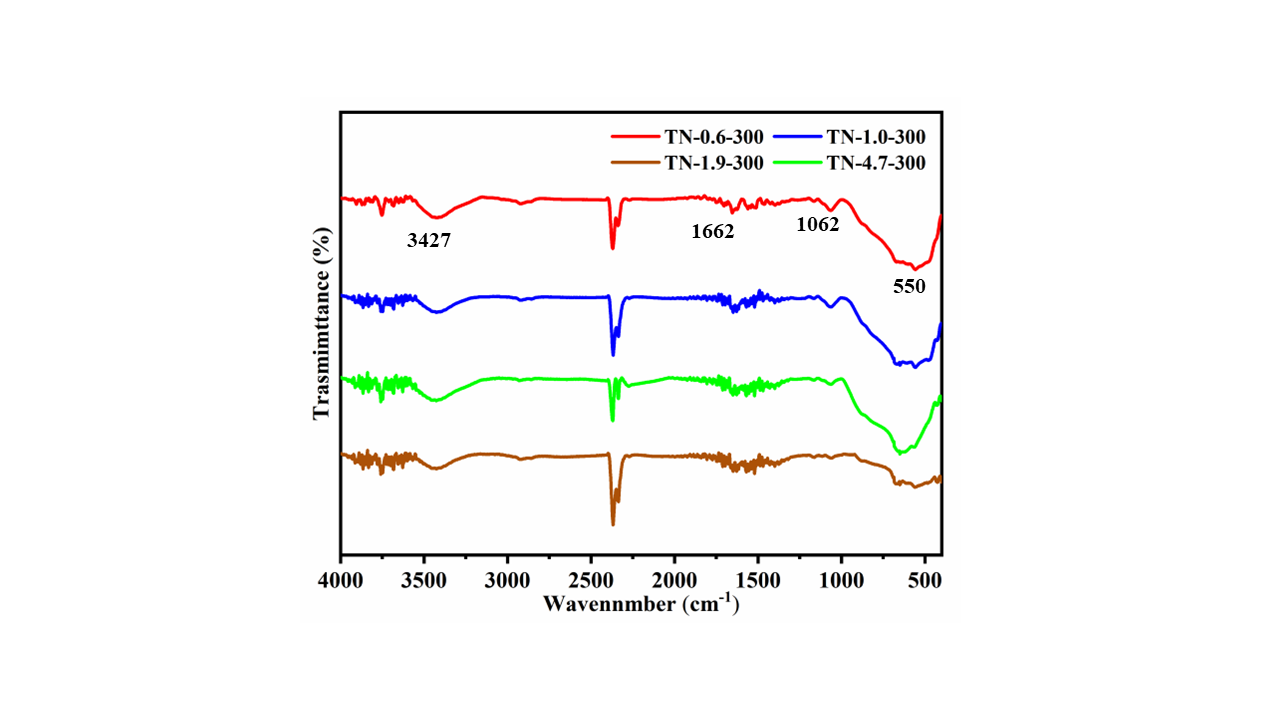


**Fig. S5.** FT-IR spectra of the as-prepared samples.


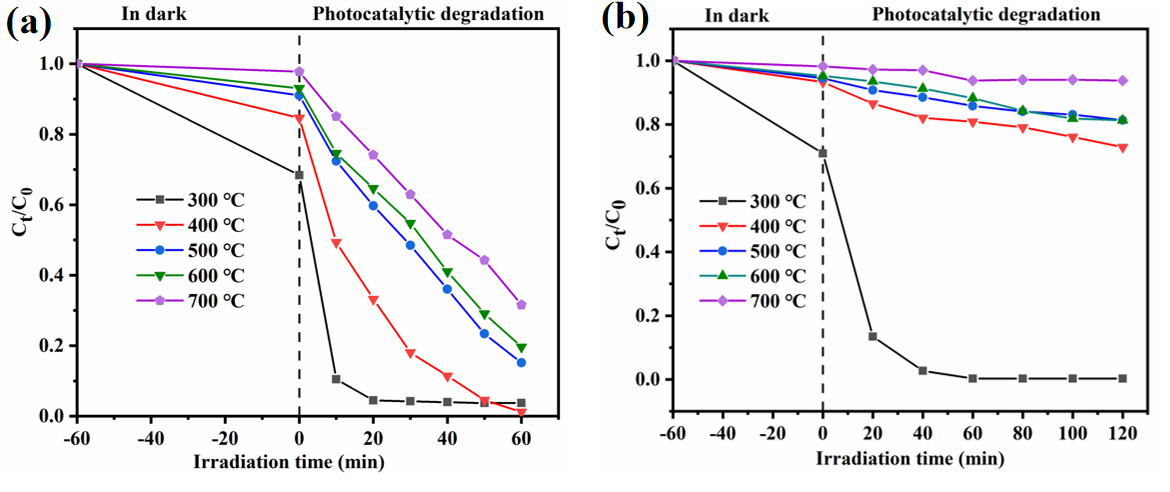


**Fig. S****6.** Photodegradation of MO by TN-1.0 prepared at different calcination temperatures (a) depicts the degradation under simulated solar radiation, while panel (b) shows the degradation under visible light radiation.

**proof of retouching a dissertation**

**
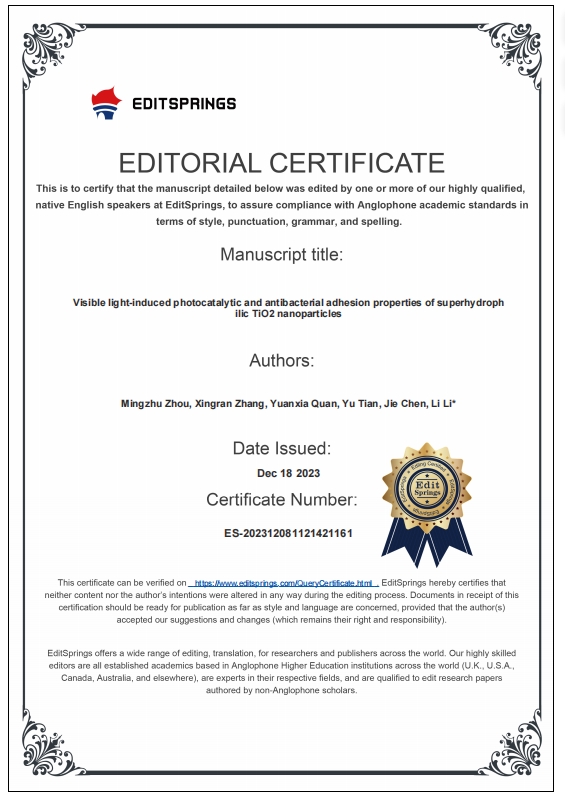
**
